# Knowledge gaps and research priorities to understand sex differences in immunity

**DOI:** 10.1371/journal.pbio.3003578

**Published:** 2026-02-02

**Authors:** Katie L. Flanagan, Sabra L. Klein

**Affiliations:** 1 Centre for Infectious Diseases and Microbiology, Westmead Hospital, Sydney, New South Wales, Australia; 2 School of Medicine, University of Tasmania, Launceston, Tasmania, Australia; 3 School of Health and Biomedical Science, RMIT University, Melbourne, Victoria, Australia; 4 W. Harry Feinstone Department of Molecular Microbiology and Immunology, Johns Hopkins University Bloomberg School of Public Health, Baltimore, Maryland, United States of America

## Abstract

Differences in immunity in males and females throughout the life span manifest as differences in susceptibility to chronic diseases, infections, cancer, and responses to therapeutic interventions such as immunomodulatory drugs and vaccines. Sex steroids and sex chromosome-linked immune response genes have major roles in driving these differences, but the cells and signaling pathways governing these are disease-specific and often not known. Such knowledge is required to better understand sex differences in disease incidence and clinical course, and to provide treatments tailored to sex-divergent pathways underlying specific diseases. This Essay explores the major areas where further research is required to determine sex-differential mechanisms.

## Abstract 2

Immunity differs profoundly according to biological sex, leading to sex differences in autoimmunity, infections, cancer and responses to immunotherapy and vaccines. This Essay explores the major areas where further research is required to determine sex-differential mechanisms in immunity.

## Introduction

Sex differences have been described across every aspect of the human condition and throughout the life span [1–4]. However, identifying the biological mechanisms mediating sex differences in human diseases is proving challenging due to the complexity and heterogeneity of the human immune system and multiple contributory and confounding factors. Advances in technology, including multi-omics assays and advanced cell phenotyping and functional analyses, allow simultaneous analysis of multiple components of the immune system to understand how they interact. Furthermore, appropriate animal models and studies of humans receiving sex hormone therapy are advancing our understanding of the relative contribution of sex chromosome-linked genes and sex steroids. Nevertheless, there are still major knowledge gaps that need to be filled before we can tailor prevention and treatment strategies based on biological sex. In this Essay, we identify key knowledge gaps across multiple sex-differential conditions that, if answered, could improve human health for everyone.

## Sex differences in immunity across the life span

Human immune systems vary broadly among healthy individuals, with most of this variation being nonheritable, as illustrated by population studies [5]. The composition of the immune system varies by sex throughout the life course, manifesting as sex differences in frequencies and function of all major immune cell subsets in both the innate and adaptive immune systems in humans [1–3]. Immunological sex differences commence in utero and persist through to old age [6]. In general, functional studies indicate greater innate and antibody responses in females than age-matched males, with differences often being most pronounced during reproductive ages [7]. This effect is nuanced and changes at different life stages; for example, immune responses during pregnancy are broadly tolerogenic with reduced inflammatory responses, particularly at later stages of pregnancy [8]. Notably, some studies contradict one another; for example, some report greater numbers of regulatory T cells (T_regs_) in females [9], while others report greater numbers in males [10]. Furthermore, some studies reporting sex differences are contradicted by others reporting no sex difference for the same parameter, for example, reports of greater [11] or equivalent numbers [9] of neutrophils in females compared to males. Some sex differences in immunity are consistent throughout the life span (e.g., greater frequencies of natural killer (NK) cells and a lower ratio of CD4:CD8 T cells in males than in females [12,13], while others vary by life stage, with many sex differences commencing at puberty, lending support to there being a hormonal mechanism (e.g., greater B cell activity in women than men [14]). Nevertheless, there is evidence that post-menopausal women continue to exhibit some sex differences in immunity (e.g., greater numbers of CD4^+^ T cells and B cell responses and reduced pro-inflammatory and increased anti-inflammatory cytokines) compared to age-matched men [15,16]. These dynamic changes inevitably drive sex differences in susceptibility and therapeutic responses to multiple diseases throughout life, a phenomenon that continues to be overlooked in modern medicine.

## Mechanisms of sex differences in immunity

### Sex steroids

**Fig 1:**
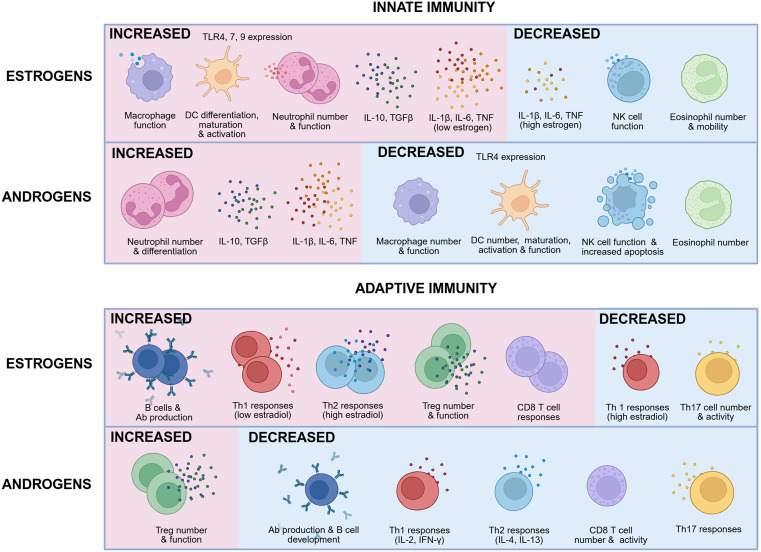
The impact of estrogens and androgens on innate and adaptive immunity. Summary of the general impact of estrogens and androgens on various components of the innate and adaptive immune systems, showing that, overall, estrogens enhance immunity while androgens predominantly suppress innate and adaptive immunity. The figure shows broad generalizations, however the effects are complex and context-dependent; for example, low and high estrogen levels have opposite effects, and androgens can have both enhancing or inhibitory effects on neutrophils. Created in BioRender. Plebanski, M. (2025) https://BioRender.com/r0oyzbi.

**Fig 2:**
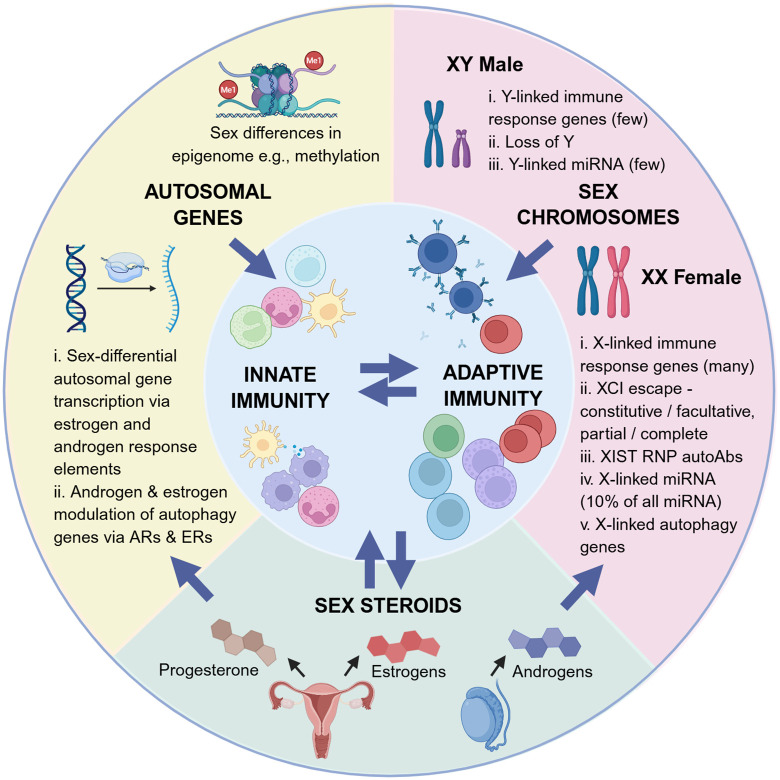
The interplay between sex hormones and autosomal and sex-linked chromosomes in driving sex differences in immunity and diseases. Both autosomal and sex-linked genes are under the influence of sex hormones via their expression of hormone response elements (HREs). Sex hormones thereby influence gene transcription, and estrogen specifically regulates expression of autophagy genes. Sex hormones also influence gene expression by causing epigenetic modifications, such as the increased methylation associated with estrogen. The X chromosome has a key role in driving sex-differential immunity via a series of mechanisms including the expression of multiple X-linked immune response genes, many of which partially or fully escape X inactivation, expression of XIST RNP, the many X-linked miRNAs and X-linked autophagy genes. The Y chromosome has a lesser role via the expression of a small number of Y-linked immune response genes and miRNAs, and loss of Y, which occurs in a mosaic fashion in some cells and increases with age. Created in BioRender. Plebanski, M. (2025) https://BioRender.com/uixfoxz.

The study of the role of sex steroids in pathogenesis is complicated by fluctuating gonadal steroid levels in males and females throughout life, particularly in women during their menstrual cycle and pregnancy [7]. Sex steroid signaling impacts both innate and adaptive immunity via the expression of estrogen receptors (ERs) and androgen receptors (ARs) in immune cells, leading to sex differences in immunity [17] (Figs 1 and 2). Many genes possess hormone response elements (HREs) in their promoter regions, specifically, estrogen or androgen response elements (EREs or AREs, respectively), which directly affect transcription [18] (Fig 2). The distribution and level of expression of sex steroid receptor proteins impact the response, and varies from one person to another. Emerging evidence indicates that periods of profound hormonal shifts, such as puberty, pregnancy, menopause, menopausal hormone therapy (MHT), and gender-affirming hormone therapy (GAHT) elicit a broad range of immunological, transcriptional, and epigenetic adaptations in blood immune cells [3,19–22].

Broadly speaking, estrogens and androgens have opposing effects on the immune system, with estrogens driving pro-inflammatory responses and androgens being anti-inflammatory [3] (Fig 1). However, the picture is far more complex than this because estrogenic effects are dose-dependent with lower doses being pro- and higher doses being anti-inflammatory. The anti-inflammatory effects of progesterone have been explored elsewhere both in the context of sex differences and pregnancy-associated changes in disease susceptibility [23–26]. The effects of gonadal steroids also vary by cell type, inflammatory context and tissue-specific factors. Importantly, pre-pubertal boys undergo a marked testosterone peak in early life and girls and pubertal women have higher circulating estrogens that peak in their twenties and thirties, both factors which drive immunological sex differences from early life [3,27,28]. While we understand many individual effects of sex steroids on immunity, it is challenging to tease out how they act in synchrony in vivo to drive sex differences in diseases and treatment outcomes.

### Sex chromosome-linked immune response genes

While sex steroids have long been implicated in driving sex-differential immunity, sex-linked immune response genes are also thought to be major contributors. The X chromosome contains multiple immune-related genes, including those involved in cellular activation and intracellular signaling (*CD40L*, *EPAG*, *TLR7*, *TLR8*, *IRAK1*, *IL13RA1/2*, *NFKBRF*, and *IL9R*), immune cell differentiation and proliferation (*IL2RG*, *BTK*, *CSF2RA*, and *ELK1*), T_reg_ development and function (*FOXP3*), leukocyte trafficking (*CXCR3*), cellular metabolism (*CYBB*), and androgen sensing (*AR*) [2,29]. In girls and women, one of the X chromosomes in each cell is switched off in a process called X-chromosome inactivation (XCI) (Fig 2). The process of XCI is regulated by a long noncoding RNA produced by the gene *XIST*, which is only expressed in healthy cells of girls and women or in those from boys and men who have an extra X chromosome, such as those with XXY Klinefelter syndrome [30]. XIST coats the X chromosome in cis and silences most genes. However, mouse experiments show that some genes (e.g., *KDM6A* and *DDX3X*) are constitutive escapees and avoid silencing in most cell types, while others (e.g., *TLR7*) are facultative escapees that only escape XCI in specific contexts [31]. The gene product XIST combines with 81 unique binding proteins in mice, many of which are known to be autoantigens in various diseases, to form an XIST ribonucleoprotein (RNP) complex [32]. Its role in sex-differential disease pathology in humans is not known, but human patients with autoimmune diseases have autoantibodies to multiple components of XIST RNP [32].

The human X chromosome contains 10% of all known microRNAs (miRNAs), small, nonprotein-coding RNAs that regulate most human genes, including those involved in immunity (118 in total compared to only 4 in the Y chromosome) (Fig 2). Some of these miRNAs escape XCI, leading to higher levels in women than in men [33]. miRNAs target 30%–50% of all protein-coding genes [18], and while their functions remain largely unknown, some X-linked miRNAs are known to affect the development and function of immune cells. For example, miR-221 regulates the survival and proliferation of human dendritic cells (DCs) and T cells [34,35], and miR-223 is involved in the regulation of granulocyte differentiation and function in mice [36,37]. Escaped miRNAs show distinct sex-differential patterns of expression in humans [38], for example, in cancers such as melanoma [39] and autoimmune diseases, particularly systemic lupus erythematosus (SLE) [40]. miRNAs have also been implicated in affecting responses to chemotherapy and radiotherapy in mice and humans [33]. However, the role of miRNAs in the development of sex differences in diseases remains relatively unexplored despite their potential as biomarkers and therapeutic targets. Future studies could analyze sex-specific miRNA expression heterogeneity in animals and humans to determine their role in driving sex-biased diseases. Such detailed studies of the role of X-chromosome immune response genes, XCI XIST and X-linked miRNAs in driving disease pathology in animal models and humans are needed to unravel some of the sex-differential etiologies of disease.

While often overlooked, the Y chromosome is also involved in gene regulation, and loss of Y chromosome (LOY), which accumulates with increasing age, occurs to varying degrees in all cell types, including immune cells, thereby contributing to sex differences in immunity (Fig 2) [41,42]. LOY causes immune cell function decline, particularly in NK cells and monocytes, leading to a more immunosuppressive state, and is associated with increased mortality in men [33]. In particular, expression of the gene encoding CD99, which is located on the Y and X chromosomes and is crucial for leukocyte function, is decreased following LOY in humans [43]. The role of the Y chromosome and LOY in the etiology of sex differences in disease processes remains largely unexplored, but the limited evidence for Y-chromosome-driven sex differences in autoimmunity, infectious diseases, and cancer susceptibility will be discussed in the relevant sections below.

### Sex-differential epigenetic control of immunity

Autosomal genes can also contribute to sex-differential immunity by being expressed differently in men and women. Epigenetic mechanisms finely regulate gene expression, and changes in the epigenome can contribute to multiple diseases. Animal and human studies report sex-specific DNA methylation and transcriptomes in circulating immune cells, suggesting that sexual dimorphism occurs at the epigenetic level [44,45] (Fig 2). Distinct changes to the epigenetic landscape occur in tandem with phases of hormonal change throughout life, such as puberty, pregnancy, menopause, and exogenous hormone therapy, implicating a role for sex hormone signaling [45]. Indeed, the formation of a sex-specific methylome during puberty containing a high frequency of EREs in humans implicates an etiological role for estrogen signaling [46]. The dynamic changes in the human blood methylome throughout pregnancy (mostly decreased methylation) mirrors changes in immune cell function involved in the shift to an immune tolerant phenotype [47]. This includes dampened pro-inflammatory innate immunity, in part mediated by increased estradiol and progesterone, human chorionic gonadotropin-driven T_reg_ development and reduced numbers of CD8 T cells, and a progesterone-mediated T_H_2 cell bias. Menopause has also been associated with methylome changes alongside marked changes in the hormonal landscape [48]. Longitudinal studies are lacking, and it remains unclear whether these methylation changes are caused by or are an effect of the changing sex hormone levels during these transition periods. Therefore, longitudinal studies analyzing the hormonal milieu alongside the immune transcriptome, epigenome, and proteome across different life stages are needed to elucidate the contribution of the epigenetic landscape to sex-differential diseases throughout life.

### Sex differences in autophagy

A key sex-differential cellular pathway is autophagy, a ubiquitous cellular process required to maintain homeostasis in which damaged organelles and obsolete cellular material are removed by lysosomal degradation [49]. It can be upregulated under conditions of metabolic stress and is altered in multiple disease processes including cancer, infections and autoimmunity [49]. Both physiological and pathological autophagy are sex-differential, with sex hormones (estrogens and androgens) and sex chromosomes implicated mechanistically. For example, experiments in rats indicate that female cells have lower basal autophagy in vitro but a greater propensity for autophagy under stress than male cells [50–52] potentially providing a survival benefit for female cells under adverse conditions. The human X chromosome encodes for several genes involved in autophagy such as *LAMP2*, which is involved in fusion between autophagosomes and lysosomes, and several RAB family GTPases involved in vesicular trafficking (Fig 2) [49,53].

Both androgens and estrogens modulate all phases of autophagy from induction to degradation, although the precise molecular mechanisms are still not fully understood and seem to be highly context-dependent. Multiple autophagy genes are transcriptionally regulated by ERα and ERβ in humans [54], supporting a key role for estrogen signaling in driving sex-differential autophagy (Fig 2). In vitro studies using human tissue or cell lines indicate that autophagy can be up- or down-regulated by estrogen responsive transcription factors such as STAT3, NFκB1, TP53, and JUN [55–58]. Thus, estrogen receptor signaling induces or inhibits autophagy under different circumstances, which is attributed to the varying expression of ERs according to cell type and physiological state. In vitro experiments in mice and humans further demonstrate that estradiol can regulate the expression of autophagy-related proteins by suppressing or stimulating the expression of miRNAs, such as miR-214 [59,60] and miR-21 [61,62]. Estradiol-mediated regulation of proteins in histone modifier complexes can also interfere with the nuclear regulation of autophagy. Estradiol-mediated activation of nitric oxide synthase 3 via human endothelial cell ERs leads to nitric oxide production, suppression of mTOR, and induction of autophagy [63]. ER distribution in lysosomes is also estrogen-dependent, but it is not known if estrogen receptor signaling has a role in lysosome mediated autophagy. Androgens can also promote autophagy via AR-mediated autophagy gene expression, a pathway which has been linked to human prostate cancer growth [64].

Determining the molecular mechanisms of dysregulated autophagy in various diseases offers great therapeutic potential. Detailed mechanistic investigation of the role of autophagy in driving sex-differential pathology is therefore needed in animals and humans, for example, knocking out different components of the autophagy pathway in animal models of disease. Furthermore, clinical trials of autophagy inducers (e.g., rapamycin, everolimus) and inhibitors (e.g., chloroquine) are also warranted in diseases where dysregulated autophagy is thought to be causative, such as those underway for the treatment of breast cancer [63].

### Sex differences in microbiota

**Fig 3:**
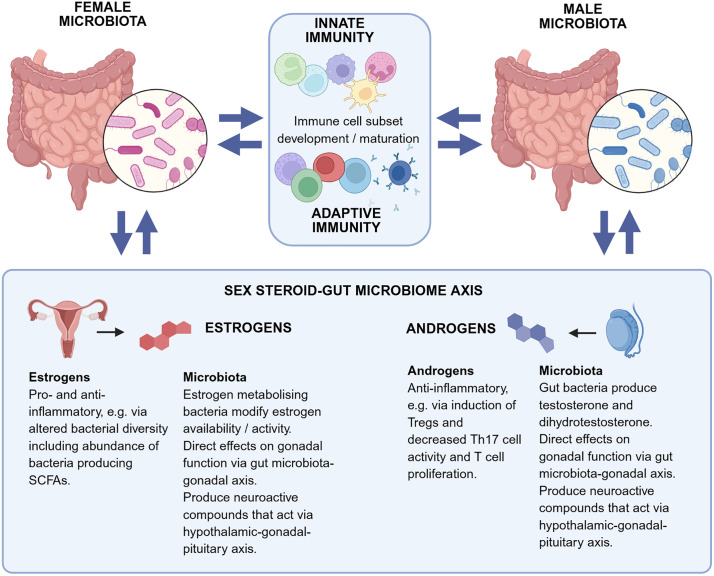
The role of the microbiota in driving sex-differential immunity and diseases. Men and women have different gut microbiota diversity and composition leading to the concept of a ‘microgenderome’ or ‘microsexome’. The sex-differential microbiota contribute to sex differences in innate and adaptive immunity via direct effects on immune cell subset development. There is a bidirectional relationship between the gut microbiota and sex hormones in what is known as the sex steroid–gut microbiome axis. Estrogens generally drive a pro-inflammatory microbiota while androgens induce an anti-inflammatory environment. Certain gut bacteria impact sex hormone levels, for example, via their ability to metabolize estrogens or produce androgens, which in turn can affect gonadal function leading to the concept of the gut microbiota–gonadal axis. Other impacts on gonadal function include the microbiota influence on the inflammatory environment and on the gonadal microbiota. Created in BioRender. Plebanski, M. (2025) https://BioRender.com/ozaxgx3.

The gut microbiota, pivotal in maintaining immune homeostasis, also contributes to sex-differential immunity via bidirectional interactions with innate and adaptive immunity and sex steroids (Fig 3). Gut microbial diversity and composition differ in males and females from birth in what has been termed the microgenderome [65] or microsexome [66] (Fig 3). Newborn human males have a lower α-diversity, lower Clostridiales abundance, and greater abundance of Enterobacteriales and Bifidobacteria than newborn females [67,68]. Further sex differences develop during puberty implicating a role for sex steroids [69,70]. A greater abundance of *Ruminococcus* and *Prevotella* has been reported in pubertal men [71,72], and bacterial α-diversity, *Bacteriodes*, and *Clostridiales* are generally greater in women [69,73,74]. However, conflicting results have been reported, possibly attributable to differences in stool processing methodology, differences in sequencing technology used (ribosomal 16s versus next generation sequencing), age of subjects, diet, and other environmental factors. Since the microbiota influences development and maturation of multiple immune cell types including innate lymphoid cells, CD4^+^ helper T cells, T_reg_ cells, B cells, and regulatory B cells, as well as IgA production [75], sex differences in the microbiota inevitably drive sex differences in immunity.

The sex steroid-gut microbiome axis describes the bidirectional relationship whereby sex steroids regulate the composition and function of the gut microbiota, while the gut microbiota alters sex steroid levels. A direct influence of sex steroids is supported by changes in the microbiota that occur at different life stages in association with hormonal shifts. For example, proteobacteria and actinobacteria abundance increase during pregnancy [76] and post-menopausal women have a more similar microbiota to men than to pre-menopausal women [77]. Other pathways involved in the sex steroid-gut microbiome axis include the biosynthesis or metabolism of sex steroids by the microbiota, regulation of systemic inflammation directly affecting gonadal function, and the production of neuroactive compounds that alter sex steroid production via the hypothalamic–gonadal–pituitary axis [78]. Certain gut bacteria, the genes of which are collectively called the ‘estrobolome’ [79], produce enzymes such as β-glucuronidase that increase estrogen bioavailability and activity [80]. It remains unclear how sex steroids modulate the colonic ecosystem to impact immunity, but some studies indicate a pro-inflammatory effect of estrogens involving the gut microbiota [81]. By contrast, androgens are thought to have anti-inflammatory activity in the gut, for example via the induction of T_regs_ which decrease T_H_17 cell activity [82] and T cell proliferation [81] (Fig 3).

Certain gut bacteria (e.g., *Clostridium scindens*) convert glucocorticoids into dihydrotestosterone and testosterone, albeit with limited impact on circulating androgen levels [83]. Testosterone can also remodel the gut microbiota composition; for example, human men with higher testosterone had a more diverse gut microbiome [72] and greater abundance of Firmicutes including Clostridiales [84] than those with low testosterone. Furthermore, the gut microbiota may also influence sex steroid levels via direct effects on gonadal function in what has been called the GELDING theory (Gut Endotoxin Leading to a Decline in Gonadal function), although this remains to be proven [85,86]. The sex differences in the microbiota increase with puberty and diminish with advancing age, particularly in post-menopausal women, supporting an etiological role for sex steroids [87].

The microbiota is implicated in driving sex differences in many chronic diseases. When estrogen-metabolizing bacteria decline, estradiol levels drop impacting the risk of certain estrogen-dependent diseases, including cancer. Experiments in germ-free, specific pathogen-free and gnotobiotic (with known colonizing microbes) animals have been used to tease out the relative contribution of microbes to sex-specific disease pathogenesis, for example, autoimmunity [88]. Nevertheless, the link between dysbiosis, sex differences in gut microbial composition and inflammatory diseases remains largely unexplored, so these studies should continue in different disease models. A deeper understanding could offer simple therapeutic approaches to treat these conditions, given the ease with which the microbiota can be manipulated.

## The effects of sex differences in immunity on disease and vaccine response

### Autoimmune diseases

Women are disproportionately affected by multiple autoimmune diseases compared to men, including SLE, autoimmune thyroid disease, rheumatoid arthritis, primary Sjögren syndrome, rheumatoid arthritis, and multiple sclerosis, among others [3]. However, men are more likely to develop type 1 diabetes, Crohn’s disease and ankylosing spondylitis than women. The factors driving this propensity for autoimmunity in women are multifactorial, with many of the above discussed mechanisms implicated, particularly sex steroids and sex-linked immune response genes. Many of these diseases become sex-differential following the onset of puberty, implicating sex hormones in the etiology. Indeed, it was widely assumed for years that sex differences in autoimmunity are largely driven by sex hormones, given the known pro-inflammatory effects of estrogens and anti-inflammatory effects of androgens. However, most studies investigating this assumption in humans are correlative only, relating disease activity with sex hormone levels and pro-inflammatory markers such as IL-6. More recent analyses confirm that multiple pro-inflammatory (e.g., TNF, IL-6, and IL-1β) and anti-inflammatory cytokines (e.g., TGF-β, IL-10) are linked to the pathology of autoimmune diseases [89,90]. Animal experiments involving sex hormone manipulation strongly implicate a role for estrogen in driving autoimmune conditions and testosterone in preventing autoimmunity [4], however the detailed mechanisms need to be determined.

A key etiological role for X-linked immune response genes in autoimmunity is well established. While the precise mechanisms remain unknown, it is largely thought to be via the biallelic expression of certain X-linked immune response genes that either fully or partially escape XCI, including *TLR7*, *TLR8*, and *CD40L* [4,91]. Mouse experiments support this notion, for example, increased *Tlr7* gene dosage promotes the development of a lupus-like disease in nonautoimmune mice; deletion of the *Tlr7* gene in lupus-prone mice reduces lupus pathology [92]; and *Tlr7.1* transgenic mice that express 8–16 extra copies of *Tlr7* develop early onset autoimmune disease [92–94]. Dysregulated XCI in female mice led to reactivation of X-inactivated genes in monocytes, DCs, and B cells, including members of the TLR7 signaling pathway, resulting in autoimmunity [95]. In humans, overexpression of *TLR7* in patients with SLE drives the expansion of transitional B cells, in which high levels of TLR7 signaling promotes auto-antibody production, linking TLR7 activity in the pathogenesis of SLE [96]. Other studies suggest that up-regulation of TLR7-mediated interferon-α (IFNα) production by plasmacytoid DCs drives the pathogenesis of SLE [97], and excessive TLR7 signaling by DCs is also implicated in the etiology of Sjögren syndrome [98]. TLR8 was aberrantly upregulated in plasmacytoid DCs accompanied by an enhanced type I IFN signature in a cohort of patients with systemic sclerosis consisting mostly of women [99], and CD40LG was aberrantly overexpressed in primary T cells and sometimes in primary B cells from women with SLE [100,101] and those with systemic sclerosis [102]. These findings implicate biallelic expression of these X-linked genes in immune cells in the pathogenesis of autoimmune disease. Another unconfirmed hypothesis is that XCI skewing leads to a mismatch between self-reactive T cells in the thymus (where they are deleted) and the presence of self-reactive peripheral T cells, which can drive autoimmunity [4].

Expression of the XIST RNP complex (containing multiple autoantigenic components) induced the production of autoantibodies and multisystem lupus pathology in male mice by reprogramming T and B cells to behave like those from females [32]. The XIST-binding RNPs acted as TLR7 ligands and promoted autoimmunity. Expansion of atypical B cells, also known as double negative (DN) B cells, was linked to XIST RNP expression in this study. DN B cells are highly sensitive to TLR7 activation and contribute to the pathogenesis of lupus in mice [95]. Given that humans with autoimmunity have autoantibodies to multiple XIST RNP components, their role in human autoimmune disease warrants exploration, as does the contribution of DN B cells to the development of autoimmunity. Indeed, DN B cell levels correlate with SLE disease severity in women [103,104]. A deeper understanding of XIST function and how its aberrant expression drives autoimmunity may identify XIST as a potential therapeutic target or biomarker to track or predict disease progression. Larger studies focusing on which XIST-related antigens contribute to female-biased autoimmunity would be of value.

Y chromosome polymorphisms have also been linked with certain autoimmune conditions in mice and humans. For example, the copy number of two Y chromosome genes, *sly* and *Rbmy*, correlates with experimental allergic encephalomyelitis and myocarditis in a mouse model, and the same applies to men with multiple sclerosis compared with healthy individuals [105]. LOY is greater in men with autoimmune thyroiditis [106] and primary biliary cirrhosis [107] than in healthy men, suggesting an etiological role.

The gut microbiota seems to have a contributory role in the pathogenesis of various autoimmune diseases [108]. Experiments in germ-free mice suggest that the microbiota contribute to sex differences in autoimmune pathologies and demonstrate crosstalk between sex hormones and the microbiota [88]. Identifying the precise microbes driving these sex differences has proved complex, but recolonization experiments in germ-free mice have implicated certain microbes, such as *Lactobacilli* as beneficial in mouse models of lupus [88]. Only certain sex-differential features of the disease were influenced by the microbiota, while others, such as splenomegaly were unaffected, suggesting alternative sex-differential mechanisms at play. Applying these findings to humans is difficult, hence most human studies describe associations rather than cause. For example, patients with SLE have different gut microbial compositions compared to healthy individuals, generally with greater *Bacteroidetes* and reduced *Firmicutes* [109,110]. Nevertheless, this is an interesting line of investigation, since manipulation of the microbiota could be explored as a simple therapeutic strategy.

In summary, the relative roles of sex steroids, XIST function, XIST RNP autoantibodies and a sex-differential microbiota in the pathogenesis of autoimmunity needs to be fully understood to devise strategies to treat autoimmune diseases that predominate in women. Longitudinal follow up of cohorts would be helpful in determining why autoimmunity develops in certain women and not others, how it is initiated and maintained, and how these effects change over the life span. Unraveling this would greatly contribute to the development of next generation therapies for autoimmunity.

### Infectious diseases

Sex differences in incidence, severity, treatment response, and outcomes have been described for multiple viral, bacterial, parasitic, and fungal infections [3,4,111]. Men and boys are affected by a greater incidence of many infectious diseases and have more severe infections and worse clinical outcomes from birth and throughout adulthood. However, this is a generalization, and in some instances, women and girls have worse outcomes (e.g., influenza A infection) [112]. It is important to remember that gendered factors such as behavior and exposure to pathogens likely contribute to these sex disparities and must be borne in mind when trying to understand mechanisms.

Proposed biological mechanisms predominantly implicate sex hormones, X-linked immune response genes and XCI, yet this is relatively underexplored, and many uncertainties remain. LOY has also been implicated, for example, in the greater severity of COVID-19 in men as compared to women [113]. Sex-differential susceptibility to certain infections commences at puberty and the severity of many infections increases during pregnancy, implicating a hormonal mechanism [114]. Sex disparities in TLR signaling have also been implicated; for example, men respond more robustly to TLR2, which is activated by gram-positive bacteria and mycobacteria, and respond better to the gram-negative bacterial sensor TLR4 [115,116]. By contrast, since the viral sensors TLR7 and TLR8 are X-linked and escape XCI under certain contexts, their expression is often greater in women [112]. TLR7/8 agonists that mimic RNA viruses trigger responses that are attenuated by testosterone therapy in individuals assigned female sex at birth [20], implicating a sex steroid role in addition to XCI effects. By contrast, lipopolysaccharide-mediated stimulation of the TLR4 receptor induces stronger responses in monocytes from the same cohort of *trans-*men during testosterone therapy as compared to baseline [20]. More studies like these will help determine the relative contribution of sex-linked genes and sex hormones.

The mechanisms mediating sex differences in infectious disease susceptibility and outcomes need to be systematically investigated. Separating the relative contribution of sex steroid receptor expression and X chromosome complement in regulating the epigenome, transcriptome, and proteome of immune cells is required. The sex-differential microbiota and sex differences in autophagy likely have a role and could be investigated in animal models and humans. Indeed, gut dysbiosis has been linked with susceptibility to several infectious diseases including hepatitis B and C, SARS-CoV-2 and tuberculosis [117]. While animal studies are helpful, they are unable to represent the environmental factors of importance in shaping human immunity, and studies of intersex and transgender people would offer a more tractable opportunity in this regard. Sex hormone biology also differs significantly between mice and humans, underscoring further the need for human cohort studies [118,119].

### Vaccine responses

Alongside sex differences in infectious disease outcomes, there are also marked sex differences in vaccine responses. Women mount greater antibody responses than men to most vaccines across the life course from childhood to old age [3,120–122]. Very few studies have analyzed for the impact of vaccination on innate immunity and results are conflicting; for example, female infants have greater innate responses compared to male infants following BCG vaccination [123], and male infants had greater innate responses following measles vaccination compared to female infants [124]. Since it is understood that vaccines can train the immune system epigenetically to cause long-term changes in innate immunity [125], further research should determine whether there are sex-differential innate immune training effects of vaccination across the life span. The impact of sex on adaptive T cell responses to immunization is even less clear, and results are also contradictory. This is in part due to sex not being considered in most human vaccine research, and most human vaccine studies being focused on antibody responses.

The more robust immunity in women comes at the cost of increased adverse events following immunization (AEFI) [15,122]. This has led to the suggestion that women may benefit from lower doses of vaccines than men to obtain the same antibody response but fewer AEFI than if the full dose was given [126]. There are exceptions to this rule; for example, men experience a greater incidence of myocarditis following mRNA COVID-19 vaccination than women for unknown reasons [127], and older men are more susceptible to adverse events following yellow fever vaccination than age-matched women [128].

As expected, sex hormones have been implicated as a major driver of the more robust antibody response to vaccines in women but may also mediate greater AEFI [8,129]. While animal studies support this, few studies have investigated this in humans. There is also scant knowledge regarding the influence of sex-linked genes in driving sex-differential vaccine responses, thus assessing the impact of XCI and LOY would be helpful. For example, aberrant expression of *TLR7* and *TLR8* from the inactive X in immune cells could explain sex differences in innate responses to viral vaccines, a question that warrants investigation, particularly since TLR7/8 ligands are being employed and further developed as vaccine adjuvants [130].

A systems vaccinology approach using multi-omics technology is being used to determine molecular signatures following vaccination that predict immunogenicity and AEFI, however few such studies have analyzed for sex differences [131]. A yellow fever vaccination study showed that women had 10-fold more differentially transcribed genes compared to men [132], and a study in African infants showed markedly different post-vaccination gene expression profiles in boys and girls following diphtheria-tetanus-whole cell pertussis vaccination [124]. The NIH/NIAID Human Immunology Project Consortium and Immune Signatures Data Resource consists of open access systems immunology data from 53 human adult cohorts and 24 vaccines offering the opportunity to interrogate for sex differences across multiple vaccines and cohorts [133]. Most systems vaccinology studies have focused on adults in high-income settings, thus future studies should include neonates and children and those residing in low- and middle-income settings.

The role of the gut microbiota in modulating vaccine responses is of increasing interest but remains underexplored. The mechanisms remain uncertain, but suggestions include natural innate stimulatory abilities, reprogramming of antigen-presenting cells, modified germinal center formation, production of immunomodulatory metabolites, and cross-reactive B and T cell responses [134]. Several studies link *Bifidobacterium* spp. with enhanced responses to certain infant vaccines, possibly via lowering inflammation and induction of regulatory B cells [134]. Indeed, *Bifidobacterium infantis*, which is associated with reduced risks of immune-mediated diseases, skews adaptive immune cell development and dampens the overall inflammatory response to commensals [135] as a likely mechanism for improved vaccine responses. Studies further suggest that antibiotic use can impair, and probiotics may improve, responses to certain infant and adult parenterally administered vaccines via an altered gut microbiota, but results are conflicting, and these questions remain unresolved [134]. Since the microbiota is different in men and women [65], any of these mechanisms could contribute to sex differences in vaccine responses.

### Cancer

Sex differences are present in the incidence and prognosis of many different cancers, which are often attributed to sex hormone effects via their ability to modulate the tumor microenvironment (TME) [136]. The impact of sex steroids is dependent on their circulating and local levels, the level of expression of sex steroid receptors by cells in the TME, and the type of tumor involved. While it is widely accepted that sex steroids, in particular androgens, have a major role in regulating tumor responses via their many effects on innate and adaptive cells in the local TME, many other sex-specific factors are at play. These include the role of sex chromosome complement, sex-differential microbiota composition, sex-differential epigenetically regulated gene expression, which can all contribute to sex differences in the TME and, in turn, impact cancer progression and responses to cancer therapies [137].

Many cancers are sensitive to sex steroids, hence blockade of sex steroid signaling is part of the therapeutic approach (e.g., androgen deprivation therapy for prostate cancer, ER antagonists for breast cancer). Recent studies using emerging tools that manipulate sex steroid levels and receptor activity in animals are identifying the specific molecular pathways involved, allowing the design of more targeted treatment approaches for malignancy [136,138]. Sex differences in autophagy mediated by ER signaling have an important role in the development and progression of many cancer types including gastric, lung and bowel cancer, and hematological malignancies [49]. Cells from multiple cancer types express ERα and ERβ, with ERα often associated with pro-tumor activity while ERβ frequently acts as a tumor suppressor via promotion of autophagocytosis and apoptosis [49]. Therefore, since estrogen signaling can both promote and diminish cancer cell growth, further in vitro and in vivo models across diseases exhibiting sex-differential autophagy are required. Elucidating the estrogen-sensitive molecular pathways involved in this process offers the potential to identify novel molecular targets to overcome treatment resistance and improve cancer survival. The sex steroid–gut microbiome axis is also thought to contribute to susceptibility to sex hormone-sensitive malignancies [86] and responses to immunotherapy [78], but studies are few and this needs further exploration.

As with autoimmune diseases, sex chromosome-linked genes are also thought to drive sex differences in malignancy. For example, many malignancies show a sex bias that has been associated with 6 genes with tumor suppressor functions that escape XCI [18,139]. Loss-of-function mutations of these genes occurs more in men, leading to their increased cancer susceptibility, while their expression from the inactivated X protects women from the cancer. There is also mounting evidence of XIST acting as both a tumor suppressor gene and an oncogene in different mouse and human cancers, noting also that XIST is somatically activated in some cancers in men [140]. For example, myeloproliferative and myelodysplastic syndrome are triggered by deletion of *XIST* in hematopoietic cells in mice [141]. In humans, prognosis was improved by XIST overexpression in patients with Hodgkin’s lymphoma [142], and XIST suppresses miR-92b, which targets Smad7 to prevent progression of hepatocellular carcinoma [143]. Evidence implicating an oncogenic role for XIST include its promotion of cell proliferation and cancer aggressiveness in human nonsmall cell lung cancer [144] and up-regulation in human gastric cancer cells, with overexpression linked to more advanced disease and knockdown having tumor-suppressive effects [145].

LOY has been strongly correlated with cancer incidence and progression in men. Specific mechanisms include aberrant activation of Y-linked *RBMY* and *TSPY* in men with hepatocellular carcinoma, and up-regulation of the Y-chromosome gene *KDM5D* in colorectal and prostate cancer [33]. LOY in T_regs_ is also thought to lead to enhanced suppressive function in the TME in colorectal, prostate, and bladder cancers [33,42].

Responses to cancer therapy also appear to be sex-differential, with women purportedly responding better to traditional chemotherapy and men responding better to immunotherapy [146]. A large meta-analysis of 10 randomized controlled trials in >11,000 people suggested that men benefit more than women from immune checkpoint inhibitor therapy, but other meta-analyses failed to replicate this finding, possibly due to concomitant chemotherapy being given or variations according to the cancer in question [4,147]. Potential mechanisms include many of the factors discussed herein. The complex interaction between sex steroids, gut microbiome, and immunity offers key target areas for improved cancer therapy since sex hormone treatments and microbiome manipulation are relatively cheap and simple. Autophagy also significantly impacts responses to cancer treatments including chemotherapy and immunotherapy [49]. Thus, autophagy-inducing immunotherapies such tyrosine kinase inhibitors and rapamycin, and autophagy inhibitors such as ATG protein blockers, have been utilized to enhance cancer treatment efficacy. It remains to be determined whether the addition of estrogens can further enhance the effects of autophagy regulators in a sex-differential manner.

Stronger immunity in women comes with the cost of greater adverse reactions to standard chemotherapy, much like the situation with vaccines. Detailed studies to determine the sex-specific molecular pathways in cancer etiology are needed to design immunotherapeutic approaches tailored individually to men and women. An in-depth understanding of the relative role of sex hormones and sex-linked immune response genes in cancer etiology would help with a precision medicine approach to treating cancers in the future.

## Experimental models to study sex differences in immunity

The human immune system is an orchestra of numerous interacting pathways, influenced by multiple factors unique to the individual including hormones, genetics, epigenetics, metabolism, diet, lifestyle, and environment. While sex steroids and sex chromosome-linked immune response genes likely have a major role in driving sex differences in many diseases and treatment responses, the predominant factors are proving difficult to decipher.

High throughput multi-omics technologies and bioinformatics pathway analysis offer great promise for unraveling this mystery and determining the key drivers of sex differences in immunity for individual diseases and therapeutic interventions. Interestingly, bulk transcriptional profiling of innate and adaptive immune cell subsets in humans showed highly cell-specific sex-differential autosomal gene expression of 1,875 unique transcripts, suggesting specificity in sex-differential gene expression [148]. One predominant feature in women is upregulated expression of genes involved in type I IFN signaling in multiple cell types [148]. A detailed analysis of the role of each of the upregulated transcripts in sex-differential immunity and disease susceptibility is therefore warranted.

The four-core genotypes (4CG) mouse model allows four genotypes to develop within a single mouse litter: XX chromosomes with ovaries, XX with testes, XY with ovaries, and XY with testes [149]. This provides a powerful tool for discriminating between the effects of biological sex (XX or XY) and gonadal sex (ovaries or testes). It is used to uncouple the effects of sex hormones and sex chromosomes to determine how they independently influence the outcome of interest. The model can also be extrapolated to humans for investigating the role of sex hormones or analogous human genes in driving sex-differential disease etiology and effects, including responses to vaccines [150].

In humans, GAHT provides a valuable opportunity to study the relative impact of sex hormones and sex chromosomes on sex-differential immunity and disease susceptibility. Multiple immunological effects have been described in line with the known immunological effects of sex hormones, including changes to innate and adaptive immune components and the gut microbiome [20,28]. Individuals undergoing GAHT can further provide unique insights into the impact of sex on disease susceptibility, and potentially help to identify new therapeutic targets. Indeed, transgender females receiving estrogen are more likely to develop SLE and lupus nephritis than their male counterparts, and cutaneous lupus improved in transgender males receiving testosterone, pointing to hormonal etiologies [45]. However, these observations are based on single case reports so do not confirm a link between the hormone therapy and development of autoimmune disease. The epigenetic effects of GAHT have only briefly been investigated and showed a potentiation of NFκB pathways in lymphocytes following gender-affirming testosterone treatment in *trans-*men [20,151]. This research area is in its infancy, and larger population studies of individuals monitored longitudinally for longer time periods and across different tissues will be important to better understand sex hormone mediated adaptation in humans [28,119].

Humans with aberrant numbers of sex chromosomes, such as Turner syndrome (XO), Klinefelter (XXY), and polysomy X (XXX), can also provide key insights into the role of sex chromosomes in driving sex differences in various diseases, and could be studied longitudinally alongside age-matched controls. For example, a study of Klinefelter individuals confirmed that the greater maturity and activation of neutrophils in women compared to men is hormonally driven rather than due to differential doses of X-chromosomes genes [152].

## Study design considerations

While many funding bodies now mandate that biological sex be investigated and reported in preclinical studies, it is still rarely reported in human clinical trials. Mice and other laboratory animals are not immunologically identical to humans, including in terms of environment or exposures, so extrapolating findings to humans may not be appropriate. When sex is reported it is often analyzed as a confounder rather than a contributory variable. This means that in a regression model, any sex difference at baseline would be the same at all levels of the predictor and may lead to the false conclusion that there are no sex differences since the effect of sex was not analyzed but just controlled for [153].

It is important to remember that sex differences refer to the biological factors that distinguish men from women, whereas societal and cultural factors that lead to differences between males and females are gender differences. Gender may therefore have a role in many of the described differences between males and females; for example, health seeking and reporting behavior, differences in occupation, recreational activities, environmental exposure, and diet and alcohol consumption. This should be borne in mind and disentangled when interpreting studies reporting sex differences, since analyses may be controlling for a combination of sex and gender.

Furthermore, for decades animal studies often used only one sex, typically males, to reduce variability in results. This means that females have historically been excluded from biomedical research studies due to their reproductive cycles interfering with results. In human studies, females have been excluded since the 1970s when concerns were raised that they might become pregnant while taking an experimental drug. Indeed, pregnant women continue to be excluded from most clinical trials, as occurred with clinical trials of COVID-19 vaccines, leading to a lack of data with which to make safe recommendations [154]. There are therefore multiple published studies in which sex was not reported or considered, and such studies are unlikely to be repeated. Consequently, women are often treated based on data derived from men, which can have negative repercussions in terms of treatment outcome and adverse events. More clinical trials involving women will be key to our understanding of sex-differential diseases and the potential role of therapeutic interventions.

## Conclusions

Males and females continue to be treated the same across most diseases and health interventions despite strong evidence showing sex differences in immunity, disease etiology and susceptibility, and therapeutic responses. More multidisciplinary research is needed to understand the basis of sex differences in immunity and their legion of effects on multiple diseases across the life span (Box 1). These need to be disentangled from gender differences, which can be difficult.

**Box 1 Major knowledge gaps and research priorities in the etiology of sex differences in diseases.**

- Sex hormone effects:
  ◦ The role and mechanisms whereby sex hormones drive sex-differential diseases and treatment outcomes.
  ◦ Studies of humans undergoing hormone-replacement therapy and gender-affirming hormone therapy to understand the relative contributions of sex-linked chromosomes and sex steroids.
- Sex-linked immune response genes:
  ◦ Detailed investigation of X-linked immune response genes known to escape X chromosome inactivation in the etiology of sex-differential diseases.
  ◦ The role of aberrant XIST expression and XIST RNP components in development of autoimmune diseases.
  ◦ The role of sex-specific miRNA expression heterogeneity in the development of sex-differential diseases.
  ◦ The role of the Y chromosome and loss of Y in the etiology of sex-differential diseases, including autoimmunity and cancer.
  ◦ Studies of humans with aberrant numbers of sex chromosomes (XO, XXY, and XXX) to determine the role of sex chromosomes in driving sex-differential diseases
- The role of epigenetics in driving sex-differential diseases, including DNA methylation of sex-linked (X, inactivated X and Y chromosomes) and autosomal genes during different life stages (e.g., puberty, pregnancy, and menopause).
- The role of autophagy in driving sex-differential diseases, particularly cancers.
- Gut microbiota:
  ◦ The causal link between sex differences in gut microbial composition, dysbiosis, and sex-differential diseases and vaccination responses.
  ◦ Ability of prebiotics, probiotics, and postbiotics to improve sex-differential diseases and vaccine responses across the life span.
- Animal studies:
  ◦ Applicability of the four-core genotypes mouse model to sex-differential diseases in humans.
  ◦ Use of humanized mouse models to understand sex-differential mechanisms.
  ◦ Systematic analysis of each X- and Y-linked immune response gene using gain/loss-of-function experiments in immune cells across various disease models.
- Longitudinal human studies across the life span:
  ◦ Ensure women are included in all clinical trials, including pregnant individuals.
  ◦ Systems biology studies analyzing for sex differences in cell-specific differential gene expression, proteome, metabolome in sex-differential diseases, treatments, and vaccination.
  ◦ Longitudinal studies of immune cell function, disease outcomes, and response to therapies and vaccines.
  ◦ Analysis for sex-differential innate immune training effects for a range of vaccines.

The advent of genomic, transcriptomic, and epigenomic profiling technology combined with sophisticated bioinformatics analysis and multi-dimensional flow cytometry is providing a more detailed understanding of the mechanisms. Ultimately, the underlying factors causing sex differences across multiple diseases are likely multifactorial and this is where knowledge is particularly lacking. The interplay between sex hormones, sex-linked immune response genes (including XCI), the epigenome and microbiota will need to be teased out in detail to determine the optimal therapeutic approach to managing sex-biased diseases in men and women throughout the life span.

Our understanding of sex-differential immune cell function mostly derives from animal experiments; however, these need to be expanded to identify detailed mechanisms and test therapies. Since the applicability of animal studies to humans remains a concern, animal models that mimic sex-differential disease patterns in humans could be used. The role of each of the X- and Y-linked immune response genes could be systematically investigated using gain- and loss-of-function experiments in immune cell populations across various disease models. In vitro studies of human immune cells in healthy people and those with sex-differential diseases are greatly needed. Longitudinal studies of immune cell function, disease outcomes and response to therapies and vaccines should be conducted across different life stages (e.g., puberty, pregnancy, menopause, and old age). Direct measurements of sex steroid levels and knowledge of sex chromosome-linked gene expression would increase our ability to understand the results of such longitudinal studies. The effects of sex hormones on the transcriptome, methylome, chromatin landscape and immune cell function all warrant further investigation in humans [7]. Individuals undergoing hormone-replacement therapy and GAHT offer a unique opportunity to better understand the relative contributions of sex hormones and sex-linked immune response genes. Future studies should aim to ensure stable T cell differentiation by limiting negative actions of sex hormones, an important step in the effective use of sex hormones as immunotherapy. Studies should also decipher the contribution of the human microbiota to sex-differential disease susceptibility, including longitudinal studies during treatment and relapses, an approach which has been taken with children at high-risk of type 1 diabetes [155].

Current knowledge gaps mean that it is not yet possible to tailor treatments and interventions by sex, potentially resulting in unnecessary harm and poorer outcomes (Box 1). Continued progress will help refine vaccine development and treatment strategies for autoimmune and other inflammatory diseases and cancers. Indeed, our knowledge base is growing, and the gaps are being filled, offering the exciting potential for improved global health outcomes in the future where everyone is treated optimally.

## Abbreviations

4CG: four-core genotypes
AEFI: adverse events following immunization
ARs: androgen receptors
DCs: dendritic cells
DN: double negative
ERs: estrogen receptors
GAHT: gender-affirming hormone therapy
HREs: hormone response elements
IFNα: interferon-α
LOY: loss of Y chromosome
MHT: menopausal hormone therapy
miRNAs: microRNAs
NK: natural killer
RNP: ribonucleoprotein
SLE: systemic lupus erythematosus
TME: tumor microenvironment
Treg: regulatory Tcell;
XCI: X-chromosome inactivation
